# Evolutionary Mismatch in Mating

**DOI:** 10.3389/fpsyg.2019.02709

**Published:** 2019-12-04

**Authors:** Cari D. Goetz, Elizabeth G. Pillsworth, David M. Buss, Daniel Conroy-Beam

**Affiliations:** ^1^Department of Psychology, California State University, San Bernardino, San Bernardino, CA, United States; ^2^Division of Anthropology, Evolutionary Anthropology Program, California State University, Fullerton, Fullerton, CA, United States; ^3^Department of Psychology, The University of Texas at Austin, Austin, TX, United States; ^4^Department of Psychological & Brain Sciences, University of California, Santa Barbara, Santa Barbara, CA, United States

**Keywords:** mating, evolutionary mismatch, evolutionary psychology, attraction, relationships

## Abstract

Evolutionary mismatch concepts are being fruitfully employed in a number of research domains, including medicine, health, and human cognition and behavior to generate novel hypotheses and better understand existing findings. We contend that research on human mating will benefit from explicitly addressing both the evolutionary mismatch of the people we study and the evolutionary mismatch of people conducting the research. We identified nine mismatch characteristics important to the study of human mating and reviewed the literature related to each of these characteristics. Many of the people we study are: exposed to social media, in temporary relationships, relocatable, autonomous in their mating decisions, nulliparous, in groups that are socially segmented, in an educational setting, confronted with lots of options, and young. We applied mismatch concepts to each characteristic to illustrate the importance of incorporating mismatch into this research area. Our aim in this paper is not to identify all potential mismatch effects in mating research, nor to challenge or disqualify existing data. Rather, we demonstrate principled ways of thinking about evolutionary mismatch in order to propel progress in mating research. We show how attending to the potential effects of mismatch can help us refine our theoretical and methodological approaches and deepen our understanding of existing patterns in the empirical record. We conclude with specific recommendations about how to include consideration of evolutionary mismatch into research on human mating.

## Introduction

Evolutionary mismatch is the idea that physiological and psychological adaptations operate in environments that differ meaningfully from the environments in response to which they originally evolved (e.g., Tooby and Cosmides, 1990; Nesse and Williams, 1994). Mismatch concepts have been addressed across a number of domains, including medicine, health, and human cognition and behavior. Our goal is to explicitly address theorizing about mismatch in one particular domain of human psychology and behavior: human mating. In this paper, we focus on analyzing the ways in which many of the people we study, and we as researchers, embody mismatched characteristics. We consider how sample and researcher mismatch can influence the generation of our hypotheses, the design of our studies, the interpretations of our findings, and ultimately our understanding of human nature. We conclude by offering recommendations for addressing and incorporating mismatch into research on human mating from an evolutionary perspective.

### Why Mating Research?

Evolutionary mismatch in research on human mating deserves analysis for three reasons. First, the enterprise of studying human mating from an evolutionary perspective is a research success story. Rigorous application of evolutionary theory to understanding mating cognition and behavior began in the 1980s and has produced an impressive body of work over the past three decades. Not only has studying human mating from an evolutionary perspective provided a foundational framework through which to understand existing research, it has generated knowledge on a host of psychological and behavioral phenomena previously unstudied or poorly understood – including mate selection criteria (Buss, 1989; Kenrick et al., 1990), sexual strategies (Gangestad and Simpson, 1990; Buss and Schmitt, 1993), mate attraction tactics (Thornhill and Gangestad, 1994; Schmitt and Buss, 1996), tactics of mate retention (Flinn, 1988; Buss and Shackelford, 1997), mate poaching (Schmitt, 2004), derogation of competitors (Buss and Dedden, 1990), jealousy-inducing qualities of mating rivals (Buss, 2013a,b), and many others (Symons, 1979; Buss, 2013a,b). The broad focus on human mating adaptations within the evolutionary social sciences is warranted. Reproduction is the currency of evolution, and successful reproduction in sexually reproducing species requires successful mating. Natural selection, therefore, will have profoundly shaped the mating psychology of all species, including humans. And because differential reproductive success is the force that drives evolution, adaptations designed to increase mating success have wide-ranging effects on behavior in many other domains, including intrasexual competition (Buss, 1988), aggression (Wilson and Daly, 1985), status-striving (Turke and Betzig, 1985; Low, 1989), and parent-offspring conflict (Daly and Wilson, 1999). The continued success of the evolutionary mating research program requires that researchers remain critical assessors of our own work. Such assessment is necessary to shape future research and to bolster the validity of existing work. Analysis of the evolutionary mismatch of the populations and samples we study provides one avenue of critical assessment.

Second, much of the existing research on human mating has been conducted on people who are likely to be mismatched from ancestral environments. This is expected, as the vast majority of living humans reside in environments that differ substantially from the likely range of conditions experienced by our ancestors (Tooby and Cosmides, 1990; Foley, 1995). Even people living in traditional cultures, such as modern foraging or horticultural populations, are living in conditions that are probably mismatched from ancestral environments. Changes in land ownership, migration patterns, trade, integration in wage markets, and access to modern technologies ranging from shotguns and chainsaws, to birth control and vaccines, to computers and the internet, have all impacted the ways in which people in modern small-scale societies live, thrive, and survive (Marlowe, 2010; Hill and Hurtado, 2017). However, there is utility in testing adaptationist hypotheses in different environments, and specifically in those environments that are more similar in important ways to likely ancestral environments, particularly in features such as group size and mobility, subsistence and fertility patterns, and the interdependence of close kin for survival (Lee and DeVore, 2017).

Pollet and Saxton (2019) empirically examined the diversity of samples described in papers published in the 2015–2016 volumes of *Evolution and Human Behavior* and *Evolutionary Psychology*, two of the leading journals that publish research on human behavior and psychology from an evolutionary perspective. They found that the majority of samples were online or student samples, and 81% of the samples came from Western cultures. Although these journals include studies on topics outside of human mating, the findings from this study support the conclusion that the preponderance of the data used to test hypotheses about human mating adaptations is derived from people living in environments that dramatically differ from the likely range of ancestral environments that shaped the very adaptations we are investigating.

Analyses of sample diversity must rely on demographic information researchers report in their published papers (e.g., participant nationality). However, researchers can better assess mismatch by identifying social and environmental characteristics that are likely to comprise meaningful mismatch. Greater specificity of mismatched characteristics allows a researcher to assess how those characteristics may or may not act as input into our evolved information-processing mechanisms. From there, a researcher can generate predictions about how the mismatched characteristic influences mechanism output, furthering our understanding of the underlying design. In this paper, we highlight nine specific characteristics of evolutionary mismatch identified by an *a priori* theoretical analysis that are especially relevant to the study of human mating. In reference to Henrich et al. (2010) work highlighting the non-representativeness (or WEIRDness: Western, Educated, Industrialized, Rich, Democratic-ness) of subjects in studies on human psychology and behavior, we arranged our characteristics into a useful acronym that modifies theirs- STRANGELY WEIRD. Our samples are often characterized by people who interact with Social media, engage in Temporary relationships, can Relocate with relative ease, have Autonomy in mate choice, are Nulliparous, experience social Group segmentation, are being tested in an Educational setting, have Lots of options, and are Young adults. Each of these characteristics represents a theoretically relevant divergence from the likely ancestral conditions under which human mating psychology was shaped by natural selection. Populations were small, the available pool of potential mates even smaller, some mate choice was heavily influenced by third-party preferences, and 20-year-olds were experienced parents (Coe and Steadman, 1995; Marlowe, 2005; Walker et al., 2011). This is by no means an exhaustive list of the domains of mating-relevant evolutionary mismatch. We focused on these characteristics because they: (1) are particularly important to the operation of human mating adaptations and, (2) provide useful examples that demonstrate the value of thinking about the implications of mismatch.

Third, because researchers who study human mating from an evolutionary perspective are themselves people who are mismatched, recognizing and evaluating mismatch may be particularly tricky. Studying psychological adaptations is inherently challenging, ironically, because of the design of the mind. The output of psychological mechanisms includes representing information about other people, ourselves, and our environment in ancestrally fitness-beneficial ways. Such representations are not tailored to provide an accurate sense of the underlying mechanisms regulating behavior and the structure of the mind (this argument has been applied to personality science, Lukaszewski, 2019). That the output of researchers’ own evolved psychology does not function to “carve nature at its joints” adds to the challenge of conducting research from an evolutionary perspective. Certain cognitive biases owing to this ancestral design, including essentialist beliefs and the appeal of teleological explanations, make engaging in an adaptationist research enterprise difficult (Tooby and Cosmides, 1990; Shtulman and Schulz, 2008). The potentially misleading effects of adaptive biases in perception and interpretation are compounded by the fact that our perspectives on human nature are ontogenetically shaped and continuously updated in the same mismatched environment in which we develop hypotheses and collect data (Fessler, 2010). Evolutionary behavioral scientists, like scientists in other fields, rely on two approaches to hypothesis generation. The theory-driven “top-down” approach consists of using existing theory to develop hypotheses about expected patterns of psychological responses or behavior. In contrast, the observation-driven, “bottom-up” approach begins with observations about patterns of psychological responses or behavior that are interpreted using existing theoretical frameworks and then used to derive novel predictions. When researchers employ this sort of inductive reasoning, they are necessarily relying on observations that occurred in mismatched circumstances. This is a critical part of any scientific endeavor, and is often the starting point for discovering novel features of human cognition and behavior. But these observations may lead researchers to draw erroneous conclusions about underlying universal design of human psychology. We hope this paper will serve as a model for explicitly considering mismatch in the process of hypothesis generation, study design, and data interpretation.

### Mismatch in Mind

Our approach to addressing mismatch is derived from the evolutionary psychologist’s model of the mind. To an evolutionary psychologist, the mind is a collection of functionally specialized information processing devices, also called evolved psychological mechanisms or psychological adaptations (Cosmides and Tooby, 1995). Each of these devices exists as it does now because, throughout its evolutionary history, it was successful in capturing some information in the environment and processing that information into affective, cognitive, or behavioral outputs that were tributary to solving some recurrent adaptive problem. Adaptive problems are recurrent obstacles to organisms’ ability to survive and reproduce, and the identification and analysis of adaptive problems is fundamental to studying evolved psychological mechanisms. Throughout human evolution, psychological mechanism variants that more effectively captured relevant environmental information, processed it according to more efficient decision rules, or produced more appropriate behavioral outputs would have more efficiently solved adaptive problems and would thereby more reliably cause the reproduction of the genes that contributed to their development. Iterated over generations, this selection process crafted in us suites of information processing systems that are improbably well-designed to solve the various reproductive problems that repeatedly confronted our ancestors. The job of the evolutionary psychologist is to recover this improbable design: to map, for any given psychological system, its inputs, decision rules, and outputs, collectively referred to as “design features”.

In one sense, this job is environment-agnostic. A complete description of a psychology will extend beyond the description of behavior in context (e.g., “bundle when cold”) and into the information processing rules that are invariant across contexts (e.g., “track current temperature in relation to ideal set point and motivate heating or cooling behaviors in response to deviation”). Simply put, if some observation varies systematically with context, it is not design *per se*, but rather the result of design. The aim, therefore, of evolutionary psychology, is not so much to map behaviors across contexts – or worse, to determine the “correct” response under the “right” conditions – but rather to elucidate, by carefully engineering available contexts, the invariant information processing design of the mind. In the same way that one does not need to see an airplane in the air to understand that it is designed for flying (its engines for thrust, its wings for flight), one does not need to see the mind under ancestral conditions to understand its many functions.

But in another very real sense, this fact also makes the environment paramount. Although evolutionary psychologists seek to understand the invariant design of the mind, the mind is nonetheless necessarily observed in a context. And that context will constrain the observations available to researchers on which to infer design. Observing the mind in just a single context – especially naïve to the mismatch between that context and an adaptations’ evolutionary history – will likely yield limited inferences. What appears like invariant design in one context may be revealed to be just one of many subroutines when observed in another; seemingly bad design could very well be good design operating under unusual conditions. An evolutionary psychologist seeking to understand the evolved design of some piece of the mind must always consider three things: (1) the adaptive problem the adaptation was designed to solve, (2) the hypothesized information processing designs that would be improbably efficient at solving this problem, and (3) the predicted ways in which this design would interact with the specific environmental contexts available to the researcher to yield empirical observations. Ignoring any of these three processes will lead a researcher to systematically flawed inferences, especially in cases where the environments in which a researcher does their science differ substantially from the environments that generated the adaptive problem in question – that is, in cases of evolutionary mismatch.

## Analysis of the Strangely Characteristics

We are not the first to discuss evolutionary mismatch in the context of mating-related adaptations. For example, Symons’s (1979) foundational work, *The Evolution of Human Sexuality*, included discussion of mismatched features of modern environments, such as pornography, sperm banks, freedom from parental influences, and potentially endless options for mates. Our goal is to build on existing research and theory that address mismatch and introduce general principles for thinking about mismatched characteristics within the domain of mating adaptations. Armed with insights gleaned from explicit consideration of mismatch related to the characteristics discussed here, we hope researchers will be better equipped to consider the implications of mismatch related to other characteristics and adaptations not described in this paper. In the following subsections, we expand on how each of the STRANGELY characteristics describes an instance of mismatch, review literature relevant to each characteristic, analyze how these characteristics may interact with our evolved psychological mechanisms, and demonstrate how researchers can incorporate consideration of these characteristics into their work. Because there is overlap between some characteristics (e.g., high rates of relocatability and social media increase the number of mate options; participants found in an educational setting are typically young) we focused on ideas unique to each characteristic in order to model different ways of thinking about mismatch and evolved mating psychology.

### Social Media

Social media, including social networking platforms such as Facebook and Instagram as well as dating platforms such as Match.com and Tinder, is a novel feature of our environment that can serve as a delivery system for cues that mimic ancestral input and affect many mating-related mechanisms. Because social media exposes people to large amounts of information in short periods of time- a user can swipe through dozens of images in minutes- it can amplify input to psychological mechanisms, especially visual input, at unparalleled levels. Interacting with people on social media, especially on platforms designed for finding mates, can provide a person with an even larger number of mate options, across a greater geographical distance, in a setting with greater anonymity than ever before. Ancestral humans residing in small groups with limited geographic mobility would have encountered, perhaps, a few dozen potential mates in their lifetime (Marlowe, 2005). Social media exposes modern humans to visual images of hundreds within a few days and many thousands over time. Importantly, even if social media does not actually alter something about a person’s real mating ecology- for example, they may never actually meet the person with whom they’ve been chatting who lives 1,000 miles away, let alone have any reproductive opportunities with that person- such cues may alter *perceptions* in ways that affect decision-making and behavior. This consideration is important both when we study online dating and mating behavior in the context of the internet, but also when we study people who interact regularly with social media.

Social media may also indirectly affect mating mechanisms by influencing self-assessment and the outputs of other psychological mechanisms, such as those designed to track status hierarchies (Wilson and Daly, 1985; Lukaszewski et al., 2016). Social networking platforms expose people to others that they may perceive as part of their social group, either as potential options for mates or competitors. Furthermore, they are exposed to others’ curated versions of themselves, designed to strategically present themselves in idealized ways (Rui and Stefanone, 2013). Self-comparison to such inaccurate portrayals may influence self-perceptions, including self-perceived attractiveness and mate value (roughly, their overall desirability in a pool of mates), which in-turn influence mating strategies. For example, men higher in self-perceived mate value score higher on measures of sociosexuality, indicating that they are more inclined toward casual sex (Clark, 2006). Self-perceived mate value is also associated with mate preferences- both men and women who are higher in self-perceived mate value place greater importance on desirable traits in potential mates, presumably because they are in a position to attract high quality partners (e.g., Buss and Shackelford, 2008; Burriss et al., 2011). Relatedly, self-perceived mate value discrepancies between a person and their partner impact mate retention tactics, relationship satisfaction, and shame in response to committing relationship transgressions (e.g., Conroy-Beam et al., 2016; Sela et al., 2017; Goetz and Maria, 2019). Thus, adaptations that regulate our self-perceived mate value, perceptions of others mate value, and our self-perceived relative standing on traits that people value in mates may be influenced by exposure to social media.

Social media usage is also positively related to anxiety and depression, and negatively related to self-esteem in adolescents (Woods and Scott, 2016). Researchers have demonstrated the same negative relationship between social media usage and self-esteem in adults, and have demonstrated that negative self-evaluations are a consequence of engaging in social comparison to people observed via social media (Vogel et al., 2014). Anxiety, depression, and self-esteem influence behavior across different types of social interactions, including romantic and sexual relationships (e.g., Shrier et al., 2001). Thus, social media exposure may affect mating outcomes indirectly through its effects on well-being.

Finally, social media provides an evolutionarily novel method through which people initiate and maintain romantic and sexual relationships- both on platforms designed for this purpose (e.g., Tinder) and on platforms with broader social networking functions. Our mate assessment mechanisms were calibrated in an environment where we evaluated potential mates in person, using the full array of cues available to us from *in vivo* interactions, observations, and third-party reports from trusted (and sometimes unreliable) sources. The information provided in an online environment is comparatively impoverished. Not only do evaluators have less information available to them- there are no scent or chemical cues, for example- but the information they do have is either declarative, or the product of selective, and sometimes deceptive, self-presentation (e.g., Toma et al., 2008). Do mating adaptations functionally assess the information about potential mates provided online as unreliable? Or in the absence of the full of array of cues to which our adaptations evolved to be sensitive, is the weak information provided online or through an app given greater weight because it is the only information available to the evaluator? Explicit mismatch framing generates these sorts of questions, and influences how we interpret findings from studies of mate selection and relationship initiation that occurs via social media platforms.

### Temporary Relationships

Evolutionary theorizing on human mating has provided a cogent framework through which to understand the variety of sexual strategies that humans pursue (Buss and Schmitt, 1993; Buss, 2013a,b). There is strong theoretical and empirical evidence that humans have engaged in committed pair-bonds across time and cultures, as well as other types of sexual and romantic relationships that vary in commitment, expectations, and time-frame (Greiling and Buss, 2000; Pillsworth and Haselton, 2006; Conroy-Beam et al., 2015; Scelza and Prall, 2018). Temporary relationships as a theoretical construct are not an example of mismatch. However, the mismatched context in which STRANGELY WEIRD people’s short-term mating adaptations operate deserves consideration. One key element of this context to consider is the degree of anonymity. STRANGELY WEIRD people who live in large societies can engage in sexual relationships with virtual strangers and, if they desire, easily never interact with that sexual partner again – a significant departure from the social environments in which our ancestors lived (Krasnow et al., 2013).

There are important hypothesized sex differences in the design of men’s and women’s short-term mating mechanism (Buss and Schmitt, 1993). In a 9-month period, an ancestral (and modern) woman could get pregnant once, compared to an ancestral man who could have produced dozens of offspring, only limited by the number of fertile women to whom he could gain sexual access. Researchers have hypothesized that men evolved a number of short-term mating adaptations as a consequence, including desire for sex with a variety of women (Symons, 1979); lowering of standards for sexual partners compared to long-term mates (Kenrick et al., 1990), and a desire to have sex sooner than women desire after first meeting a mate (Buss and Schmitt, 1993). These short-term mating adaptations are now operating in an environment with unprecedented anonymity and potential options. Male university students in particular are surrounded by many women displaying cues to youth and fertility, key determinants of judgments of female attractiveness (Kenrick and Keefe, 1992; Li et al., 2002). The combination of these cues- anonymity, many fertile options- may act as a supernormal stimulus, triggering men to pursue short-term mating strategies more strongly than they would have ancestrally. One benefit to researchers is that this makes university men a useful population to study the design of short-term mating adaptations.

Although ancestral women would not have accrued the same fitness benefits by pursuing many sexual partners as men did, researchers have hypothesized that there were likely other fitness benefits to short-term mating that shaped the design of women’s short-term mating adaptations (Greiling and Buss, 2000; Pillsworth and Haselton, 2006; Sacco et al., 2012). Women may have gained resources, such as food, protection, or status through short-term sexual relationships. Genetic benefits for offspring are another possible benefit. Additionally, some of the benefits of engaging in short-term mating may have been linked to long-term mating. Short-term mating could have allowed a woman to assess new potential partners, either because she did not currently have one or was considering leaving her current partner; could have helped her eject her current mate or switch to a better mate (Buss et al., 2017); could have helped her clarify her long-term mate preferences and practice her mate attraction skills; or could have been used as a tactic to manipulate her current mate into increasing commitment.

These differences in men’s and women’s short-term mating adaptations already set the stage for sexual conflict. However, we hypothesize that the mismatched environment of STRANGELY WEIRD people increases the likelihood and magnitude of some forms of sexual conflict. Some men primed by the mismatched environment may sexually pursue women to the point of harassment- or worse, they may employ coercive and exploitative strategies (Goetz et al., 2012). Men who are unsuccessful in gaining short-term sexual access are not only operating in an environment that strongly triggers short-term mating motivation, but they also may be attuned to others’ relatively greater success. Incels are a subgroup of men who define themselves by their inability to gain sexual access to women (incel = “involuntarily celibate”). Incels express an entitlement to sex and resent that they have been denied, display extreme misogyny toward women, and advocate for violence against women and against men who are sexually successful (Ging, 2017). We hypothesize that elements of mismatch fuel the rise of such subgroups- in addition to being primed to pursue short-term mating, incels can use online forums to discuss their ideas and develop as a group with complete anonymity.

Women’s short-term mating adaptations can also be assessed through the lens of mismatch- particularly adaptations that are hypothesized to link short-term mating motivation to long-term mating goals. Prior to contraception, engaging in a temporary sexual relationship would have been reliably linked to conception. This could motivate a woman to attempt to convert a short-term mate into an investing mate, particularly in an environment where a woman’s family and kin are nearby and could exert influence to ensure a man invests and commits. Other potential benefits to short-term mating in women, such as obtaining high-quality fish, meat, or other resources, switching to a new mate, or maintaining a potential back-up mate, are less likely to be realized if a woman’s short-term mate can more easily leave or avoid committing- which characterizes relocatable men in large societies with increased anonymity. If women’s short-term mating adaptations are blind to these novel features of our environment, then women may still feel motivation to pursue short-term mating but experience dissatisfaction with the consequences. Studies show that women are more likely to regret having had sex with someone, compared to men who are more likely to regret having missed sexual opportunities, and women are more likely than men to experience negative emotions after engaging in “hooking up” (Lambert et al., 2003; Galperin et al., 2013). We hypothesize that at least some of women’s regrets and negative experiences with short-term mating may come from environmental mismatch that results in them being less likely to gain the expected benefits from casual sexual encounters than they would have been likely to gain ancestrally.

### Relocatable

Humans, as primates, have occupied large territories and home ranges and pursued a generally nomadic lifestyle, ranging from brief seasonal relocations to nearly continuous movement across the landscape throughout our evolutionary history. Data from modern foraging populations suggest that territory sizes range from small territories of less than 300 square miles in environments with dense and reliable resources, to greatly expanded territories that can stretch as far as 1,500 square miles in desert environments (Cashdan et al., 1983). These territory sizes mark the outer limits of a group’s normal movements, primarily on foot, throughout the year. Marlowe (2005) has suggested that even relatively small home ranges in rich environments during the Late Pleistocene were probably on the order of about 110 square miles, far larger than those occupied by other primate species (by comparison, the largest territory occupied by extant chimpanzees is on the order of only about 13 square miles; Herbinger et al., 2001). However, these ranges are trivial compared to the distances that STRANGELY WEIRD people can travel with far less effort and risk. Modern transportation allows us to travel thousands of miles in hours, crossing mountains, continents, and oceans with ease. Geographic mobility in ancestral populations would not have provided even a fraction of the potential social opportunities represented by this level of mobility. In addition, the size of a foraging population’s territory is generally negatively correlated with population density (Cashdan et al., 1983), so even those covering very large geographic territories have far more limited social novelty than in dense modern environments. The experience of complete social relocation, moving alone or with only one’s immediate kin, to a large, anonymous, and entirely new community with few or no existing social bonds, would likely have been a rare event in such populations.

Such relocatability may alter perceptions of the costs and benefits of engaging in a number of mating-related behaviors, thus affecting a number of different mating-related adaptations. Behaviors that deviate from social norms, provoke retaliation, or have negative reputational consequences may be less costly if one can easily relocate. Mate poaching, the process of attempting to attract a romantic partner away from their current mate, provides an example of a mating-related behavior that can provoke retaliation and negatively affect reputation. Schmitt (2004) demonstrated that mate poaching was more common in world regions with more resources and more common in individuals higher in socioeconomic status. Although Schmitt argued that this was evidence that in less resource-rich environments, fidelity and biparental care are more important, an alternative explanation is that people with greater resources can more easily relocate and escape the negative consequences of poaching or being poached, increasing the frequency of this as a mating strategy.

Adaptations related to sexual exploitation provide another example in which the perception of unprecedented ability to relocate may have important implications. Are both men’s and women’s mechanisms related to sexual exploitation responsive to cues of extreme relocatability present in our current environment? One possibility is that men’s mechanisms that motivate sexually exploitative behavior produce behavior flexibility in response to their ability to relocate. Unprecedented relocatability could then produce a corresponding increase in sexually exploitative behavior in men, indicating these psychological mechanisms are sensitive to the extreme range of this cue that exists in our modern environment. Even if men’s psychological mechanisms are responsive to modern relocatability, women’s mechanisms to protect themselves from sexual exploitation may not be sensitive to modern relocatability if ancestral relocatability was not correlated with women’s on average greater ability to protect themselves from sexual exploitation. Another hypothesis is that neither men nor women are sensitive to the degree of modern relocatability because it did not exist ancestrally. If so, sexually exploitative behavior in men exposed to modern relocatability cues may not differ compared to men existing in an environment where cues to relocatability are similar to ancestral cue levels. Alternatively, both men and women’s mechanisms may respond in ancestrally fitness-beneficial ways to cues to relocatability, despite the fact that this cue exists at greater intensity than ever before. Studies of women’s fear of sexual assault do support the hypothesis that women’s fear adaptations are sensitive to cues related to having relocated. Ferraro (1996) found that housing tenure (the length of time a woman had lived in her current residence) was negatively associated with fear of rape in women ages 18–34. Younger women who have more recently moved may be geographically isolated from family and social networks that ancestrally would have provided protection from sexual assault. Modern female university students may experience the extreme, mismatched version of having relocated if they have traveled hundreds of miles away to attend a university. We hypothesize that university women’s fear of sexual assault may be stronger because of the extreme lack of cues of local protective family and kin. Relocating may have an even greater impact in the modern environment because of the extreme distances women may move.

### Autonomous

People in our samples are often characterized by substantial autonomy in their mating decisions, a feature that is much less common in extant small-scale societies (Apostolou, 2007). Although ancestral human groups were likely characterized by a much lower average coefficient of relatedness than other primate groups, due to bisexual philopatry and dispersal, estimates from modern forager populations indicate that a child is likely to be related to approximately 50% of the adults in a band, at the level of 2nd cousins to her parents, with an additional 25% of the adults in the band being more distantly related (Hill et al., 2011). As adolescents, the stage in life when most individuals will begin to make mate choices, a person would be surrounded by aunts, uncles, grandparents, siblings, cousins, and in-laws. In contrast, many of the STRANGELY WEIRD people we study lack immediate contact with any kin at all, and live in a population of thousands or tens of thousands of unrelated, single, young people similarly unencumbered by closely watching kin.

There is evidence that kin, and parents in particular, have evolved psychology designed to influence offspring mate choice and have historically exercised, or attempted to exercise, that influence (e.g., Apostolou, 2012). Researchers have demonstrated that parents have preferences for who their child selects as a partner, and that these preferences sometimes differ from their child’s preferences. Parents tend to emphasize a potential in-law’s social and economic resources that can benefit the family group, while offspring emphasize the individual benefits that a potential mate can bring to the reproductive bond, in particular, genetic quality (e.g., Buunk et al., 2008; Perilloux et al., 2011; van den Berg et al., 2013). Arranged marriage is the norm across many small-scale subsistence populations. Even in groups in which arranged marriage is not practiced, parents are likely to have a great deal of influence as girls are almost universally married before they are 18 (Murdock et al., 2008). Although there is evidence that parental control in small-scale populations tends to weaken with age, the attempt at parental control would likely have been a consistent feature, particularly for young women entering a mating market and embarking on their first marriage.

Thus, many of the people we study now are exercising unprecedented autonomy in their mate selection. Candidate adaptations that are important to consider with respect to unprecedented autonomy include mate selection and relationship satisfaction adaptations. STRANGELY WEIRD people may make mating decisions, including who they select as a long-term mate, that do not reflect ancestral adaptiveness because the adaptations motivating these decisions lack sufficient input from parents and kin, who ancestrally would have exerted influence. Another possibility is that lack of input promotes indecision, or delays in long-term pair-bonding in people we study whose mechanisms evolved to incorporate parental assessment or approval as input. Census data tracking age of first marriage in the United States supports this hypothesis. Contrasted with the typical age of marriage cited above in modern small-scale societies, the median age of first marriage in the United States has increased over time and is at an all-time high of 29.8 years for men and 27.8 years for women (U.S. Census Bureau, 2018). Such delays may have downstream effects, including individuals having fewer offspring or being less likely to ever marry. Alternatively, reduced parental and kin influence could result in greater mate or relationship satisfaction in STRANGELY WEIRD samples because mate choice is a product of individuals’ preferences alone, without having to compromise on their desires to satisfy family members.

### Nulliparous

Nulliparous women and childless men are not an evolutionary novelty. However, the circumstances under which people are childless in our modern environment differs from ancestral childlessness. Based on data from modern foraging societies, ancestral women were likely to have had their first child by about age 19 (Robson et al., 2006), and most sexually active couples would have conceived eventually (Bailey and Aunger, 1995). We can compare this to current birth statistics- in the United States alone, the average maternal age at first birth steadily increased from 24.9 in 2000 to 26.3 in 2016 (Centers for Disease Control and Prevention, 2016). Ancestral nulliparity would have indicated that a woman either was not sexually active, or if she was, would indicate infertility, either with her, her partner, or the combination of the two of them (for a discussion of causes of childlessness reported in the ethnographic literature, see Betzig, 1989). Modern nulliparity, and childlessness in men, is often a conscious choice in our mismatched environment where some people precisely plan when and how to procreate.

One intuitive hypothesis about nulliparity or childlessness is that we would expect childless couples to be lower in relationship satisfaction. Relationship satisfaction is hypothesized to be an internal regulatory variable that tracks the fitness costs and benefits of remaining in or leaving a long-term relationship and functionally motivates relationship maintenance or dissolution behavior (Conroy-Beam et al., 2015). It is reasonable to hypothesize that whether or not a union had produced children could have evolved to be one input into relationship evaluation adaptations. Modern infertility statistics indicate that about 9% of men and 11% of women of reproductive age have experienced fertility problems, and similar rates ancestrally could have provided selection pressure to shape adaptations to motivate leaving a relationship when the couple was infertile (Chandra et al., 2013). However, studies of relationship and marital satisfaction in modern people do not support this hypothesis. One meta-analysis of studies of marital satisfaction found that parents report lower satisfaction than non-parents (Twenge et al., 2003). Additionally, studies that focus specifically on couples who explicitly desire children but are experiencing infertility have been inconclusive. Some demonstrate decreases in women’s marital satisfaction associated with infertility, but others have demonstrated better marital functioning among infertile couples compared to fertile couples (Luk and Loke, 2015). One study even found that infertile couples experienced greater feelings of commitment and loyalty and emotional intimacy (Drosdzol and Skrzypulec, 2009).

These findings demonstrate that to test hypotheses about how nulliparity or childlessness will influence mating adaptations, their modern properties must be considered. Childless adults may be able to devote more of their energy and resources toward other pursuits- acquiring more resources, expanding social networks, increasing their status- that parents cannot because of the burdens of childcare (Shenk et al., 2016). This may demonstrate another instance of mismatch, where ancestrally more children would have been associated with increases in resource acquisition ability, expanded social networks, and increases in status (Crittenden, 2009; Wiessner, 2009). To the extent that their partnership improves their ability to pursue these alternative goals, nulliparous women and childless men may experience increases in relationship satisfaction that outweigh any negative effects of childlessness. The cue of a relationship having not produced a child may provide only one piece of input into mating adaptations, therefore; testing whether or not it is a cue requires controlling for other possible inputs that may mask its effects.

Interestingly, there is evidence that cross-culturally, infertility does often lead to divorce (Betzig, 1989). There are two possibilities for explaining why recent data about infertility and relationship satisfaction does not fit with historical data about infertility and divorce. Recent studies about relationship satisfaction could represent a change in how people in Western societies perceive childlessness. Not having children may be less of a cause for relationship dissolution than it was before, another way in which people we study may be mismatched. Alternatively, once a couple has children, divorce may be less desirable because of the potentially negative effects it could have on offspring, motivating couples to stay together even if they are less satisfied with one another. Thus, interpreting these findings, and what they mean for the underlying design of psychological mechanisms that motivate relationship dissolution, requires thinking about the mismatched elements of the people studied.

Whether or not a person already had children, and the degree of parental investment existing children required to survive and to gain competitive advantage in their sociocultural context, could have altered the fitness consequences of mating decisions, and mating mechanisms may have evolved to be calibrated by the presence and status of existing offspring (Goetz, 2016). Because women ancestrally had the greater obligatory parental investment (Trivers, 1972), we should expect parenting status to particularly impact women’s psychology. For example, mothers faced the adaptive problem of obtaining a mate who did not pose a threat to her existing children- a problem not faced by nulliparous women (Daly and Wilson, 1985). Children may also influence mating cognition indirectly through their effect on parents’ mate value, and women’s mate value in particular. Men in the United States report being less willing than women to marry someone with a child, and in the Kipsigi in Kenya, grooms’ families offer a lower bride-price for women who already have a child by another man (Borgerhoff Mulder, 1988; Goldscheider and Kaufman, 2006). This combined evidence suggests that having children negatively impacts women’s mate value, likely more than men’s mate value. Mate value has been hypothesized to influence a variety of adaptations, including mate preference mechanisms and those regulating and relationship maintenance behavior (Buss and Shackelford, 2008; Edlund and Sagarin, 2010; Starratt and Shackelford, 2012). Studying these nuances in the design of mating adaptations requires samples that vary in offspring number. Additionally, prior to contraception, the majority of a person’s mating career would have occurred when they were already parents. Therefore, studies of that include only nulliparous women and childless men may only capture a narrow slice of the design of mating adaptations.

### Group Segmentation

Ancestral bands were limited to about 150 adults who regularly interacted with one another, and who were likely to be related in some way either by blood or marriage. Not only did everyone know each other, but they intimately understood each other’s lives- the challenges, the labors, the entertainments, the daily, seasonal, and annual routines, and the random natural or social events that interrupted those routines- because they shared them (Kelly, 1995). Additionally, although there were divisions of labor by age and sex and variations in skill and specialization, everyone had some familiarity with many of the skills and tasks necessary for survival, including tool-making, food production, and caregiving. One of the consequences of such interconnectedness is that there is a high degree of consensus in small-scale populations regarding the relative social status and the particular strengths and weaknesses of others in the community: who is the best hunter, who is the best mother, who has the best garden, who is the strongest fighter, who is the worst liar, who is the biggest cheater (Bird et al., 2001; Gurven and von Rueden, 2006; Pillsworth, 2008; Escasa et al., 2010). In addition, there is strong consensus about the value of these various traits, including which traits matter most in a reproductive partner. In a Shuar community, for example, there was almost 100% consensus among men that whether a woman makes good chicha (manioc beer) is more important than if she was a virgin before marriage (Pillsworth, 2008). Even if specific community assessments are not very accurate (for example, hunting reputation among the Hadza does not appear to be a particularly good predictor of actual hunting returns, Stibbard-Hawkes et al., 2018), the reproductive consequences of one’s reputation are important, and likely reflect mating-relevant underlying qualities (Apicella et al., 2007; Apicella, 2014; Smith et al., 2017).

However, in STRANGELY WEIRD contexts, people are members of numerous, non-overlapping, sometimes highly specialized, social groups that we may shift rapidly between. In a 12-h period, a STRANGELY WEIRD person can go from a new job where they are the lowest in status and influence, to a game with their long-standing recreational softball team where they are the lead hitter, to post-match drinks at a bar among strangers. A person’s reputation and standing among one social group may not be known to those outside that group. Additionally, characteristics that result in status gains or desirability among one group may not have the same influence among a different group. Although many of the characteristics that are desired in mates- physical attractiveness, kindness, intelligence- are likely perceptible across contexts, certain traits might be expressed more within one group compared to another, and one’s relative standing on each trait could shift from group to group.

The potential consequences of this mismatch are important for researchers studying adaptations that function to track relative social valuation, mate value, and desirability of the self and others. A person’s mate value depends on their relative standing on the multitude of traits that people assess when selecting a mate (Conroy-Beam et al., 2016). Modern social group segmentation allows us to test hypotheses about the extent to which our self-perceived mate value updates when we rapidly shift from one social group to another. Furthermore, there is evidence that the diversity of social roles available to STRANGELY WEIRD people facilitates specialization and differentiation in personality (Lukaszewski et al., 2017), opening up new dimensions of mate evaluation. The existence of multiple groups likely has different consequences for men and women. Because physical attractiveness is a more important component of women’s mate value compared to men, for example, women’s mate value should change less from group to group compared to that of men (e.g., shifting from a coed campus study group to a coed softball game).

Additionally, social group segmentation may make it easier for people to shield undesirable or negative information about themselves from potential mates because their behavior and attitudes expressed in one group are unknown to those in a different group. Both men and women engage in deceptive tactics to attract mates that involve exaggerating their desirable qualities and masking their undesirable qualities (e.g., Tooke and Camire, 1991). The mismatched characteristic of extreme group segmentation allows us to test the extent to which adaptations motivating deception are sensitive to this mismatched context in which deception may be more effective, and we can test if there are increases in deception in mating when people’s social groups are more segmented. Similarly, extreme group segmentation provides researchers the opportunity to study the design of deception-detection adaptations in contexts where information about others may be more limited than it would have been ancestrally. One possibility is that because deception-detecting adaptations did not evolve in an environment in which social groups were so segmented, they fail to detect deception as well in contexts where a person’s interactions with the deceiver are limited to a particular isolated social group.

### Educational Setting

University student samples likely deviate from both non-university samples and ancestral populations in important ways. Students are surrounded by many mate options, as well as many mating competitors, who are similar in age and socioeconomic status to themselves. Although university students are often legally classified as adults and may live apart from their families, many of them remain financially and emotionally reliant on their parents. Additionally, many universities are now female-biased at the undergraduate level, and operational sex ratio is related to human mating patterns (e.g., Schmitt, 2005; Stone et al., 2007; Kruger and Schlemmer, 2009). A surplus of women in the mating pool, for example, tends to shift mating behavior more toward short-term mating, such as “hookups” and “friends with benefits” (Buss, 2016). Each of these features of university students may be important to consider from the perspective of mismatch and are cases where input may be of a level of intensity outside the range ancestral humans ever encountered.

Researchers should consider how students in their studies are interpreting questions asked of them in a research setting, and how their behavior may be shaped by their context. Li et al. (2013) argued that previous research using speed-dating methods and university samples may have not replicated sex differences in mate selection because those study designs lacked mate options who represented low-end variation on key traits like financial prospects and physical attractiveness. In one of their studies, they demonstrated that a relatively low status college student was rated as low in earning prospects when participants evaluated this person among a group of other college students. However, when the same person was evaluated among a group that contained low status individuals from the general population (e.g., a non-college graduate working at a mall), the low status college student was rated as having average earning prospects. Across three studies, Li et al. (2013) demonstrated that sex differences in mate preferences are reflected in mate selection in university students when the study design includes the full range of variation on sex-differentiated preference dimensions. These studies demonstrate that when we study college students, the reference group they are using to make comparisons matter. Outcomes will differ if they are restricting their evaluations to other university students, compared to if they are considering the population as a whole. They also reveal the importance of considering the mismatch of the people and contexts studied. Li et al. (2013) criticized the design and data interpretations of previous studies precisely because those studies did not take into account the evolutionary mismatch of the university students and setting.

### Lots of Options

STRANGELY WEIRD people have potentially endless options for sexual and romantic partners. Ancestrally, humans lived in small groups, with average band sizes estimated to not surpass a maximum of about 300 total people, including children under the age of 15, who typically make up nearly 50% of a forager group (Jones et al., 1992; Marlowe, 2005). Such bands might occasionally come together into much larger tribal units of more than 2,000 individuals, but several lines of evidence suggest that humans cognitively track only about 150 individuals (Dunbar, 1993; Hill and Dunbar, 2003). The pool of potential mates was even further limited to currently available reproductive-age adults of the preferred sex. Many of the people we study live in unprecedentedly massive metropolitan areas, where even “small” cities can contain more inhabitants than our ancestors would have met in a lifetime. Our research participants are constantly exposed to novel people, and have access to a large, people-dense, geographic radius of accessible mates.

Researchers have demonstrated that mating behavior and cognition differ depending on the number of potential partners being evaluated. Lenton and Francesconi (2010) compared “small” speed dating events, where participants met with 15–23 potential partners to “large” speed dating events (24–31 potential partners). They found that visible cues, like height and weight had a stronger influence on mate choice at large events, while non-visible cues, like occupational status and education had a larger influence at small events. Experimental research in which female participants evaluated four, 24, or 64 mate options has demonstrated that women report using different heuristics depending on option number. Women selecting one profile from four were more likely to report using a weighted average strategy where they evaluated trade-offs across attributes within a profile. Women evaluating larger sets were more likely to use an elimination-by-aspects strategy, where they eliminated options by evaluating across profiles one attribute at a time (Lenton and Stewart, 2008). Other research has addressed satisfaction with mate choice decisions when there are numerous options. D’Angelo and Toma (2017) found that online daters who chose from 24 options were less satisfied with their choice compared to those who chose from six options. Furthermore, those who chose from the larger set *and* were given the option of reversing their decision 1 week later were the least satisfied. In general, these studies demonstrate that when participants are making mating decisions in contexts in which they have many options, they think and behave differently. When options are more limited people: (1) do assess the non-visible characteristics that typically come up in studies of mate preferences, (2) rely on heuristics that involve assessing potential partners holistically across a variety of attributes, and (3) are more satisfied with their mating decisions and less subject to “choice overload” effects (Iyengar and Lepper, 2000). These findings support the hypothesis that our adaptations are sensitive to variation in the number of available partners.

However, the number of options available to many people we study represents an input into mating mechanisms that is several orders of magnitude more intense than ancestral humans were likely to have experienced. An open question is how this supernormal stimulus interacts with our mating adaptations. Do these adaptations simply ramp up their outputs in response to the novel stimulus? Or are other decision-making mechanisms co-opted in our modern environment to sort through our options in ways that would not have occurred ancestrally? Alternatively, because our mating mechanisms were shaped in environments with so many fewer options, are there limits to how responsive we should expect mating behavior to be to this unprecedented option number? Even if there are endless options, perhaps people do not *perceive* their world as though there are – and, worse for them, they may use decision strategies better suited to a bygone past, rather than strategies that are tailored to the decision problem they are actually facing.

### Young

Youth is not an evolutionary novelty; however, Western young adults differ in many ways from ancestral people in their late teens-20s. Data from hunter-gather groups indicate that children begin hunting and gathering in early childhood and are contributing substantially to a family’s caloric needs in adolescence, compared to modern children and adolescents who often do not contribute at all to family livelihood, or only minimally (Hawley, 2011; Crittenden et al., 2013; Konner, 2017). Menarche in women in hunter-gather groups occurs in the late-teens, compared 11–12 years of age in modern, Western girls (Konner, 2017). Sexual behavior is typical in adolescent hunter-gatherers, and adults exert only weak control over adolescent sexuality (Konner, 2017). Additionally, STRANGELY WEIRD children are often segregated by age, limiting interactions, socialization, and learning from older and younger individuals (Hawley, 2011). Overall, by the age of 18, ancestral individuals likely already developed adult skills related to subsistence, interacted with group members across all ages, developed some reputation and social standing, engaged in sexual behavior, if female were likely married, and may already have had children.

In contrast, modern Western young adults are just learning the ropes of self-sufficiency, many are experiencing their first sexual relationships, they often are not parents, and are in a novel extended period of skills and career building. The behavior we observe in STRANGELY WEIRD young adults may more accurately reflect mating behavior in ancestral adolescents rather than their same-age counterparts. The mismatch in age of menarche is particularly interesting. Ancestrally, sexual exploration in adolescence would have been less costly to pre-menarchal girls. The potential costs are higher in modern Western girls, who on average have fewer years of cognitive and social development to guide their decision-making prior to the onset of reproductive capability (Coe and Steadman, 1995).

Evolutionary thinking does provide a foundation for making subtle, sex-differentiated, predictions about how age should relate to mating cognition and behavior. For example, women’s reproductive window is limited compared to men’s and their probability of conceiving peaks when they are in their mid-twenties and tapers off until menopause. One of the starkest sex differences in adaptive problems would have occurred when men and women were in their mid-40s through 50s, when women experienced menopause. The Grandmother Hypothesis suggests that menopause was adaptive in women because they would have experienced greater fitness gains from investing in current offspring and current or future grand-offspring than by continuing to reproduce (Hawkes et al., 1998). Ancestral men would have experienced almost the opposite selection pressure- their status and resource holdings could accrue with age, providing them with more mating opportunities. Among the Tiwi of northern Australia, for example, men under age 30 rarely have enough status to attract a wife (Pilling and Hart, 1960). Young people are, and ancestrally were not, the only people engaged in mating-related behavior and decision-making. Studies that focus exclusively on young adults limit our ability to test age-dependent design features of adaptations. And there is a danger in testing and retesting hypotheses about universal design on a homogenous subgroup of individuals because doing so provides a narrow slice of information and may not reflect the range in variation in human mating behavior across the lifespan predictable from evolutionary theory.

## Discussion

Although each mismatched characteristic requires unique analysis, there were commonalities in our approach across characteristics. Existing ethnographic research, and research from anthropologists, behavioral ecologists, biologists, and psychologists has developed our understanding of the features of humans’ ancestral past. We drew on this interdisciplinary research to develop an understanding of how each particular characteristic embodied evolutionary mismatch. We then considered how an environment mismatched on a particular characteristic would influence functioning of specific mating-related adaptations. Fully understanding mismatch requires both a keen understanding of ancestral conditions and an appreciation of the design of information-processing mechanisms that regulate behavior. This process led to novel hypotheses that a researcher could investigate that would address human mating adaptations through the study of STRANGELY WEIRD people.

We focused on STRANGELY WEIRD people; however, even non-WEIRD people are mismatched from ancestral conditions-many now have access to smart phones, buses and other forms of transportation, and can purchase food and supplies. The approach outlined in this paper should not be limited to the study of university undergraduates, but it important regardless of the particular population being studied. Additionally, we focused our analysis on the domain of human mating, but much of what we have argued here can and should be applied to research in other areas of human psychology and behavior as well, such as cooperation, coalitions, status hierarchies, and kinship. Even the characteristics we identified may also be important to consider for those studying other types of social relationships, interactions, and perceptions.

### Organizing and Expanding on Mismatched Characteristics

We focused on just nine mismatch characteristics, but these represent just a small fraction of the many potentially important ways in which modern environments differ from the ancestral environments that forged our mating psychology. Another important mismatch not considered in detail, for example, is the absence of small-group warfare that characterized small-group living throughout much of human evolutionary history (e.g., Ghiglieri, 1999). Such warfare would have had profound effects on mating. These include mate acquisition through offensive raids and higher male than female mortality, in turn creating a sex-ratio imbalance of a surplus of women. This imbalance, in turn, may have created conditions fostering polygyny. In short, the absence of small-group raids and war in the modern environment renders it highly discrepant from ancestral environments – one of many additional mismatches affecting human mating that we have not considered in detail.

These nine mismatch characteristics can also be arrayed on higher order dimensions of mismatch, which may facilitate identifying other important mismatch characteristics. Li et al. (2018) have provided a starting point for considering the broader dimensions of mismatch. They generated four dimensions along which mismatch phenomena can be arrayed: source, type, consequences, and causes. *Sources* can be natural or human-generated. They argue for two *types* of mismatch- “forced” occurs when a new environment is imposed on an organism and “hijacked” are when novel stimuli are favored by mechanisms over stimuli that would have existed ancestrally, to which the mechanism originally evolved in response. The *consequences* dimensions involves defining mismatch phenomena on their mismatched consequences for an organisms’ fitness and/or well-being and values compared to ancestral environments. Finally, they argued that the *causes* of mismatch are either changes in input into a psychological mechanism (input may be more or less intense than it was ancestrally, entirely missing in the modern environment, or novel cues may mimic ancestral cues), or changes to the consequences of the output of an adaptation.

Many of the STRANGELY characteristics can fit easily into their dimensional framework. For instance, Relocatable, Autonomous, Group segmentation, Educational setting, and Lots of options are extremes on the causes dimension in that they represent unprecedented intensity of input to which individuals are exposed. Social media provides novel cues that may mimic ancestral cues. We also offer another dimension of mismatch phenomena to consider- mismatch between the modern properties of particular characteristic and the ancestral properties of that characteristic. Temporary relationships, Nulliparous, and Young are characteristics that require this type of consideration. Organizing along other dimensions may be more useful in other circumstances. For example, researchers specifically focused on the implications of mismatch on individual well-being may place greater focus on the consequences dimension and a helpful reviewer of this paper suggested other dimensions of mismatch. One dimension suggested by the reviewer was a “similar-different in information conveyed by a cue” dimension. The advent of cosmetic surgery, for example, may result in facial wrinkles providing less information about an individuals’ age (and, perhaps, more information about their resources) than facial smoothness would have provided ancestrally. No single dimensional framework is likely to offer optimal resolution for clearly identifying all mismatch characteristics that are relevant for a particular research question. Preferred dimensions or types may depend on the researcher’s goals and this dimensional space should evolve over time as researchers continue to test mismatch hypotheses.

### Mismatch and the Variability of Human Behavior

Research on human mating from an evolutionary perspective has demonstrated how evolved psychology produces behavioral flexibility and variability. Not all observed characteristics unique to the people we study require mismatch framing. Many modern, culturally specific behaviors and products of our behavior can be understood as instances of evoked culture, and behavioral flexibility and cultural differences are expected to occur as functional output of evolved psychological mechanisms (Tooby and Cosmides, 1992). However, these processes also contribute to generating environmental mismatch. Our evolved psychology has produced social media and devices on which to consume it, fostered the development of large, anonymous societies, and created medical technologies that allow us to precisely plan when we will reproduce. But understanding how these novel features of people and environments interact with our adaptations requires careful consideration. Their influences on both the people studied and the researchers studying them should be examined.

Some characteristics generated open questions about adaptation design that only become testable *because* of sample mismatch. For example, any adaptation that tracks option number evolved in an environment where people were exposed to fewer options than they are now. The benefits of studying samples mismatched on this characteristic is that we can determine if adaptations respond differently when option number varies outside a range that would have existed ancestrally- or if they do not. In this way, mismatch is not a barrier that prevents us from being able to establish evidence of universal psychological design. Instead, mismatch can be a useful tool.

Using a mismatch perspective also reminds researchers that the output of adaptations observed in modern people may seem curiously “maladaptive” if we attempt to assess the output’s current fitness costs and benefits. Outside the mating domain, our evolved food preferences adaptations provide a simple example. In an ancestral environment of greater food scarcity without refrigeration and grocery stores, motivation to opportunistically consume high-calorie foods would have been functional. The design features of our food preference adaptations, shaped by that environment of potential scarcity, now motivate many of us to eat far more than is necessary in our mismatched, food-rich environment, to the detriment of our health and longevity. We expect that the output of mating adaptions in our mismatched modern environment may produce similar outcomes. Consider relationship satisfaction, an adaptation hypothesized to be sensitive to the presence and number of potential high-quality alternative mates (Conroy-Beam et al., 2016). Ancestrally, lower relationship satisfaction in response to better options, possibly motivating leaving a current mate, may have been adaptive. However, in our modern environment of perceived abundance of high-quality mate options, such an adaptation could produce never-ending relationship dissatisfaction and difficulty maintaining pair-bonds. This is part of why focusing on current adaptiveness of behavior is inappropriate to understand the underlying design of our psychology (Tooby and Cosmides, 1990; Confer et al., 2010). Researchers interested in the mating behavior that results in distress, negative emotions, and seemingly dysfunctional outcomes may be able to better understand those phenomena by employing mismatch concepts.

Some of the adaptations and mating behavior we discussed relate to the darker sides of human mating- including mate poaching, sexual conflict, and rape. Our analysis sheds light on particular features of our modern environment, such as relocatability and anonymity, that we predict could increase such phenomena. Researchers interested in designing interventions to reduce these societal ills can benefit from thinking about mismatch and how evolved mechanisms respond to cue levels present in our modern environment.

### Recommendations and Conclusions

Mismatch thinking is complicated. So are the potential implications of evolutionary mismatch. Although we discussed each mismatch characteristic separately in this paper, any researcher employing mismatch concepts will practically find themselves evaluating and framing research questions with respect to multiple characteristics. Case in point, we described and hypothesized about incels in the subsection on temporary relationships, but that discussion also would have been appropriate when discussing social media. Many psychological and behavioral phenomena of interest should be considered with respect to multiple mismatched features simultaneously- which may lead to competing hypotheses. For example, we hypothesized that modern relocatability may increase sexual conflict, but the absence of small-group warfare in STRANGELY WEIRD people could have the opposite effect on sexual conflict. We even posed competing hypotheses generated by considering a single characteristic- autonomy in mate choice may decrease relationship satisfaction if influence from kin increases satisfaction, but mate choice autonomy could increase satisfaction if it is associated with less compromising of preferences. No single study is expected to capture the full complexity of any adaptation, or test all relevant mismatch hypotheses. But we are optimistic that the growing body of researchers who use an evolutionary perspective to study human mating will achieve great strides in unraveling the complicated nature of mating adaptations by engaging in the complicated endeavor of mismatch thinking.

We provide four general recommendations to facilitate the development of research programs that address mismatch. These recommendations are intended to build on the high-quality work that has been conducted on human mating from an evolutionary perspective. Researchers have made great strides in explaining the underlying universal design of mating-related adaptations, including those related to mate preferences, romantic and sexual jealousy, incest avoidance, and mating strategies (e.g., Schmitt, 2005; Lieberman et al., 2007; Buss, 2018; Buss and Schmitt, 2019). The arguments presented here do not discount existing research. We acknowledge that much of this research has been conducted in accordance with our recommendations. We offer these insights as a model for researchers going forward with the hope that a more explicit focus on mismatch will be one of the tools researchers of human mating can use to better develop and refine their work.

First, we suggest incorporating mismatch concepts into existing best-practices on how to conduct research using an evolutionary perspective. As evolutionary psychology and related fields have developed over time, researchers have elucidated how to conduct research from an evolutionary perspective. They have addressed all stages of the research process, from hypothesis generation, to study design, to data interpretation (e.g., Barkow et al., 1992; Lewis et al., 2017). Adding consideration of mismatch of the intended study subjects, particularly at the study design and data interpretation stages, should become standard practice. Researchers should consider their own mismatch throughout the process to identify instances where their own mismatched circumstances may bias their thinking.

Second, we identified nine characteristics that we believe are particularly important to consider in the field of mating behavior and psychology. These are intended to provide a starting point, but not an exhaustive list, for elements of mismatch to consider. We hope others will expand and refine this list (e.g., small-group warfare as another good candidate). Certain characteristics may be more important than others to address depending on the specific mating-related adaptations being studied.

Third, we will improve our research by proposing and testing hypotheses explicitly about mismatch when possible. Cross-cultural research, and comparisons with small-scale societies are ideal in some cases, but even within Western societies it may be possible to examine populations that vary on the identified mismatched characteristics. If it is not possible to specifically incorporate mismatch into study hypotheses or design, researchers should include these ideas in the discussion of their results. This will improve our science over time and pave the way for future research that may be better able to address mismatch.

Fourth, we suggest highlighting when a mismatched sample may actually be beneficial for testing a particular hypothesis. Characteristics that generate unprecedented levels of input into mechanisms, or result in the absence of input, are necessary to assess the range of input to which mechanisms are responsive. Effortfully addressing the benefits of a particular sample also should assist researchers in employing appropriate caution in interpreting what data from that sample does tell us about the universal design features of psychological adaptations.

To conclude, we advocate for the explicit consideration of sample and researcher mismatch throughout the research process when studying mating-related adaptations and anticipate that this will propel progress in our understanding of human nature.

## Author Contributions

CG developed and conceived the central ideas, structure of the manuscript, and had principle writing responsibility. EP and DC-B contributed to the original concept and writing. DB provided substantive feedback and revisions on all sections of the manuscript. All authors contributed to the manuscript revision, read, and approved the submitted version.

## Conflict of Interest

The authors declare that the research was conducted in the absence of any commercial or financial relationships that could be construed as a potential conflict of interest.
